# Schlafen 11 further sensitizes BRCA-deficient cells to PARP inhibitors through single-strand DNA gap accumulation behind replication forks

**DOI:** 10.1038/s41388-024-03094-1

**Published:** 2024-07-03

**Authors:** Hiroshi Onji, Sota Tate, Tomohisa Sakaue, Kohei Fujiwara, Shiho Nakano, Miho Kawaida, Nobuyuki Onishi, Takashi Matsumoto, Wataru Yamagami, Takashi Sugiyama, Shigeki Higashiyama, Yves Pommier, Yusuke Kobayashi, Junko Murai

**Affiliations:** 1 Department of Obstetrics and Gynecology, Ehime University Graduate School of Medicine, Toon, Ehime Japan; 2 Department of Biochemistry and Molecular Genetics, Ehime University Graduate School of Medicine, Toon, Ehime Japan; 3 Division of Cell Growth and Tumor Regulation, Proteo-Science Center (PROS), Toon, Ehime Japan; 4 Department of Cardiovascular and Thoracic Surgery, Ehime University Graduate School of Medicine, Toon, Ehime Japan; 5 Division of Physiological Chemistry and Metabolism, Graduate School of Pharmaceutical Sciences, Keio University, Minato-ku, Tokyo, Japan; 6 Laboratory for Metabolomics, RIKEN Center for Integrative Medical Sciences, Yokohama, Kanagawa Japan; 7 Division of Diagnostic Pathology, Keio University Hospital, Shinjuku-ku, Tokyo, Japan; 8 Department of Clinical Diagnostic Oncology, Clinical Research Institute for Clinical Pharmacology and Therapeutics, Showa University, Shinagawa-ku, Tokyo, Japan; 9 Department of Plastic and Reconstructive Surgery, Keio University School of Medicine, Shinjuku-ku, Tokyo, Japan; 10 Department of Obstetrics and Gynecology, Keio University School of Medicine, Shinjuku-ku, Tokyo, Japan; 11 Department of Oncogenesis and Tumor Regulation, Osaka International Cancer Institute, Chuo-ku, Osaka, Japan; 12 Developmental Therapeutics Branch and Laboratory of Molecular Pharmacology, Center for Cancer Research, National Cancer Institute, NIH, Bethesda, MD 20892 USA; 13 Department of Obstetrics and Gynecology, Institute of Medicine, University of Tsukuba, Tsukuba, Ibaraki Japan; 14 Institute for Advanced Biosciences, Keio University, Tsuruoka, Yamagata, Japan

**Keywords:** Tumour biomarkers, Targeted therapies, Ovarian cancer, DNA replication, Single-strand DNA breaks

## Abstract

The preferential response to PARP inhibitors (PARPis) in BRCA-deficient and Schlafen 11 (SLFN11)-expressing ovarian cancers has been documented, yet the underlying molecular mechanisms remain unclear. As the accumulation of single-strand DNA (ssDNA) gaps behind replication forks is key for the lethality effect of PARPis, we investigated the combined effects of SLFN11 expression and BRCA deficiency on PARPi sensitivity and ssDNA gap formation in human cancer cells. PARPis increased chromatin-bound RPA2 and ssDNA gaps in SLFN11-expressing cells and even more in cells with BRCA1 or BRCA2 deficiency. SLFN11 was co-localized with chromatin-bound RPA2 under PARPis treatment, with enhanced recruitment in BRCA2-deficient cells. Notably, the chromatin-bound SLFN11 under PARPis did not block replication, contrary to its function under replication stress. SLFN11 recruitment was attenuated by the inactivation of MRE11. Hence, under PARPi treatment, MRE11 expression and BRCA deficiency lead to ssDNA gaps behind replication forks, where SLFN11 binds and increases their accumulation. As ovarian cancer patients who responded (progression-free survival >2 years) to olaparib maintenance therapy had a significantly higher SLFN11-positivity than short-responders (<6 months), our findings provide a mechanistic understanding of the favorable responses to PARPis in SLFN11-expressing and BRCA-deficient tumors. It highlight the clinical implications of SLFN11.

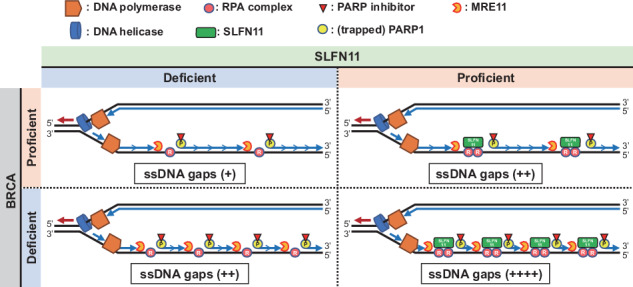

## Introduction

Poly (ADP-ribose) polymerase (PARP) inhibitors (PARPis) were the first drugs to highlight the concept of synthetic lethality in clinical oncology [1]. Initial models proposed that catalytic PARPis prevent the repair of single-strand DNA (ssDNA) breaks that are subsequently converted into double-strand DNA breaks (DSBs) upon collisions with replication forks and need to be repaired by homologous recombination (HR) [2, 3]. This explains why PARPis induce synthetic lethality in breast cancer gene (BRCA)-deficient tumors with HR defects. Based on this initial model, PARPis have been found selectively toxic to cancer cells harboring *BRCA1 or BRCA2* (*BRCA1/2*) mutations or showing HR deficiency. Although this model has proven applicable when comparing PARPi sensitivity in BRCA1/2-deficient and HR-proficient cells at relatively low concentrations (e.g., 100 nM olaparib) [4, 5], PARPis exhibit antitumor effects, even in HR-proficient tumors, in a concentration-dependent manner at relatively high doses (e.g., 1–25 μM olaparib) [6–8].

A decade ago, we discovered an additional mechanism of action of PARPis, which accounts for their antitumor activity as a single agent above the drug concentrations required for PARylation inhibition and which is referred to as “PARP-trapping” [6, 7]. We found that PARP1 and PARP2 are trapped by PARPis on DNA, generating toxic PARP-DNA complexes. Accordingly, PARPis act as “PARP-poisons” because the killing of cancer cells by PARPis completely disappears in PARP1- and PARP2-deficient cells [6]. Importantly, the potency of PARP-trapping is widely different among PARPis, with talazoparib being the strongest, olaparib and niraparib being moderate, and veliparib the weakest. Because PARP-trapping is increased by the addition of alkylating agents, e.g., methyl methanesulfonate (MMS) or temozolomide, which generate DNA base damage, we proposed that PARP-DNA complexes are generated at the intermediate steps of base excision repair. Moreover, because olaparib and MMS combination [6] or talazoparib alone [9] block DNA replication, we also proposed that PARP-trapping causes replication blocks and exerts replication stress and consequent cell killing [10]. Because PARP-DNA complexes lead to the formation of DSBs with bulky PARP proteins upon fork collision, cancer cells engage multiple repair factors beyond BRCA1/2 to manage PARP-trapping [10–12]. Thus, PARP-trapping can damage HR-proficient cells while it causes more severe damage in HR-deficient cells. Consequently, the clinical indications of PARPis have expanded beyond *BRCA1/2* mutations [13] and PARP-trapping is acknowledged as a primary mechanism of action for the anticancer activity of PARPis [14].

Recent studies have proposed that the accumulation of ssDNA gaps behind replication forks due to impaired Okazaki fragment processing (OFP) is a primary genotoxic lesion promoting PARPi sensitivity [15–18]. However, the mechanisms through which ssDNA gaps accumulate under PARPis treatment are still poorly understood, and it is unclear whether PARP-trapping is involved in the impaired OFP. Nevertheless, extensive studies have demonstrated that PARP1 is activated in the process of OFP [15] and that PARP-trapping at unligated Okazaki fragments under PARPis is essential for inducing ssDNA gaps behind replication forks [16, 18]. Furthermore, PARPis have been shown to produce excessive ssDNA gaps behind forks in BRCA1- or BRCA2-deficient cells because BRCA1 and BRCA2 are involved in OFP [17]. The nuclease meiotic recombination 11 (MRE11) has also been implicated in generating extended ssDNA gaps behind replication forks in BRCA1-deficient cells [16]. Notably, PARP inhibition does not suppress DNA replication fork rates [18], suggesting two effects of PARPis: 1/ PARP-trapping ahead of replication forks at base damage lesions causing replication blocks, and 2/ PARP inhibition causing ssDNA gaps behind replication forks, which do not arrest the DNA polymerase replicative complexes.

Schlafen 11 (SLFN11) is an emerging factor in cancer chemotherapy as it sensitizes cancer cells to a broad range of DNA-damaging anticancer agents causing replication stress, such as topoisomerase I (TOP1) inhibitors (camptothecin, indenoisoquinolines), TOP2 inhibitors (etoposide, doxorubicin), ribonucleotides inhibitors (hydroxyurea), antimetabolites (gemcitabine, cytosine arabinoside) and platinum-derivatives (cisplatin, carboplatin) [19–22]. Drug resistance due to lack of SLFN11 expression has been established in clinical as well as basic research through substantial studies from various independent laboratories [23, 24]. SLFN11 has a replication protein A1 (RPA1)-binding domain [25] and is colocalized with chromatin-bound RPA1/2/3 (RPA) complexes [25, 26]. It also directly binds ssDNA [27]. Although the mechanisms of SLFN11-mediated cell killing deserve further studies, one prominent process leading to cell death is the SLFN11-dependent replication block, which usually occurs within 4 h after drug treatment [26, 28]. The ongoing model is that SLFN11 is recruited to replication forks under replication stress where the replicative helicases and polymerases are uncoupled, which causes the formation of extended RPA-coated ssDNA that recruits SLFN11 to irreversibly arrests replication.

SLFN11 also confers hypersensitivity to PARPis in pre-clinical models [9, 29, 30], and a recent clinical study analyzing the effects of SLFN11 on olaparib sensitivity in patients with ovarian cancer showed that high SLFN11 expression is associated with improved clinical outcomes [31]. Notably, subgroup analyses revealed that only patients with both *BRCA* mutations and high SLFN11 expression benefited significantly from olaparib treatment. Because SLFN11 functions without regard to BRCA status in a variety of settings under replication stress [9, 22], we wished to elucidate the mechanism by which PARPis act when SLFN11 is expressed in BRCA deficiency cancer cells.

## Results

### SLFN11 enhances the sensitivity of BRCA1/2-deficient cells to PARPis

To examine the reciprocal effects of BRCA1/2 and SLFN11, we employed the SLFN11-expressing ovarian endometrioid adenocarcinoma TOV-112D and medulloblastoma DAOY cell lines in their *SLFN11-*knockout (SLFN11-KO) counterparts, which we recently shown to be hypersensitive to camptothecin (CPT) and cisplatin [32, 33]. Deleterious mutations in *BRCA1* and *BRCA2* are absent in either cell line, according to the Cancer Cell Line Encyclopedia database (https://discover.nci.nih.gov/rsconnect/cellminercdb/) [34].

**Fig. 1:**
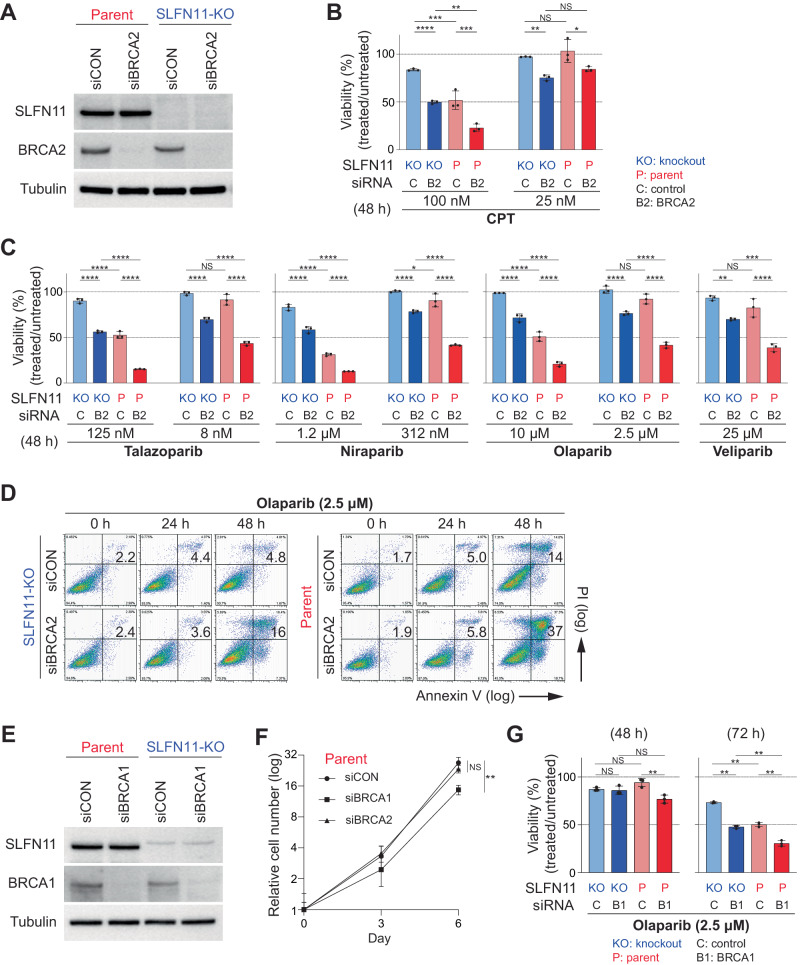
SLFN11 enhances cellular sensitivity to PARP inhibitors in BRCA1/2-deficient cells. **A** Immunoblots of whole cell lysates from genetically modified TOV-112D cells: SLFN11-proficient (parent), SLFN11-KO, control siRNA (siCON) or BRCA2 siRNA (siBRCA2). Blots were probed with the indicated antibodies. **B**, **C** Viability of TOV-112D cells under each condition after 48 h of continuous drug treatment. Cellular ATP activity was used to measure cell viability. The viability of untreated cells was set as 100%. Data are means ± standard deviations (*n* = 3, technical replicates) and represent one of two independent experiments. **D** Apoptosis analysis of TOV-112D cells by flow cytometry. The indicated cell lines were treated continuously with 2.5 μM olaparib for 0, 24, or 48 h. Data represents one of two independent experiments. **E** Immunoblots showing BRCA1 knockdown by siRNA for BRCA1. Data are shown as in **A**. **F** Cell proliferation curves of the indicated TOV-112D cells. **G** Viability of TOV-112D cells under the indicated conditions after 48 and 72 h of continuous olaparib treatment. NS not significant, *P < 0.05, **P < 0.01, ***P < 0.001, ****P < 0.0001 (one-way analysis of variance with Tukey’s post-hoc multiple comparisons test).

After knocking down (KD) *BRCA2* expression using a mixture of siRNAs in parent and SLFN11-KO cells in each cell line (Fig. 1A and S1A), we compared four conditions, presented in the following order in Fig. 1 and S1: SLFN11-KO/control siRNA (siCON), SLFN11-KO/BRCA2 siRNA (siBRCA2), parent (SLFN11-proficient)/siCON, and parent/siBRCA2. In cellular viability analyses with 48 h continuous 100 nM CPT treatment (Fig. 1B, left), TOV-112D SLFN11-KO/siCON cells were the most resistant. SLFN11-KO/siBRCA2 cells or parent/siCON cells were more sensitive than SLFN11-KO/siCON cells, and parent/siBRCA2 cells were the most sensitive. Under 25 nM CPT (Fig. 1B, right), SLFN11-KO/siBRCA2 cells were more sensitive than SLFN11-KO/siCON cells, but parent/siCON cells were as resistant as SLFN11-KO/siCON cells. Parent/siBRCA2 cells were as sensitive as SLFN11-KO/siBRCA2 cells. These results demonstrate that SLFN11 expression and BRCA2 deficiency additively enhance sensitivity to the topoisomerase I (TOP1) poison CPT.

We next examined the impact of BRCA2 and SLFN11 on the activity of the four clinical PARPis: talazoparib, niraparib, olaparib, and veliparib. At relatively high concentrations (125 nM talazoparib, 1.2 µM niraparib, and 10 µM olaparib), we observed additive effects of *BRCA2*-KD and SLFN11 expression in cell viability assays (Fig. 1C). By contrast, at relatively low drug concentrations (8 nM talazoparib, 312 nM niraparib, and 2.5 µM olaparib) and at 25 µM veliparib, where the effects of SLFN11 expression were marginal in parent/siCON cells compared with SLFN11-KO/siCON cells, the effects of SLFN11 expression were significant in parent/siBRCA2 cells compared with SLFN11-KO/siBRCA2 cells (Fig. 1C). We obtained comparable results with the DAOY cell set (Supplementary Fig. S1B, C). Additionally, flow cytometry revealed an 11% increase in dead cells in SLFN11-KO/siBRCA2 cells, a 9% increase in parent/siCON cells, and a 32% increase in parent/siBRCA2 cells compared to SLFN11-KO/siCON cells (Fig. 1D). These results establish that SLFN11 further enhances the activity of PARPis in the cells lacking functional BRCA2.

To generalize these results, we performed parallel experiments by knocking down *BRCA1* and examining the combinational effects of BRCA1 inactivation and SLFN11 expression (Fig. 1E). However, the reduced proliferation of *BRCA1*-KD cells compared to their WT counterparts (Fig. 1F) rendered it difficult to assess a potential difference in cell viability 48 h after olaparib treatment. Yet at 72 h, we observed additive effects of *BRCA1*-KD and SLFN11 expression (Fig. 1G). These results collectively demonstrate that SLFN11 enhances PARPis sensitivity in BRCA1- and BRCA2-deficient cells. To circumvent any issues arising from the slow proliferation of *BRCA1*-KD cells, we primarily focused on analyzing the effect of BRCA2 in the rest of the study.

### SLFN11 expression and BRCA2 deficiency increase chromatin-bound RPA2 in cells treated with PARPis

**Fig. 2:**
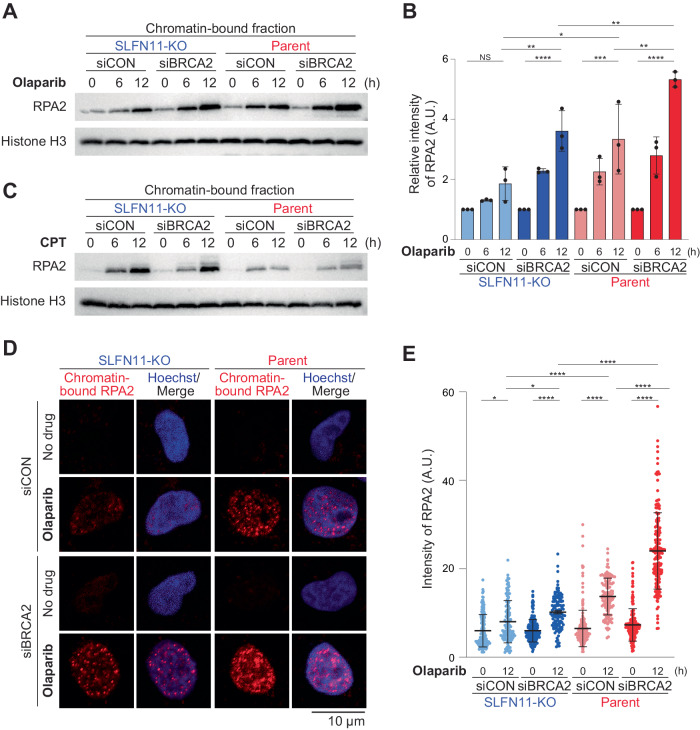
SLFN11 expression and BRCA2 deficiency increase chromatin-bound RPA2 under PARPi treatments. **A**, **C** Representative immunoblots of chromatin-bound fractions prepared from the indicated TOV-112D cells treated with 10 μM olaparib or 100 nM CPT for 0, 6, or 12 h. Blots were probed with the indicated antibodies. **B** Quantification of data from **A**. Data were normalized to the untreated cells (0 h). Data are means ± standard deviations (*n* = 3, biological replicates). **D** Representative confocal microscopy images; chromatin-bound RPA2 (red) and Hoechst (blue) in TOV-112D cells. Cells were treated with or without 10 μM olaparib for 12 h. **E** Quantification of data from **D**. Scatter plots show the mean signal intensity of RPA2. Data are means ± standard deviations (*n* = 116–199, one-time experiment). Data represents one of two independent experiments. NS not significant, *P < 0.05, **P < 0.01, ***P < 0.001, ****P < 0.0001 (one-way analysis of variance with Tukey’s post-hoc multiple comparisons test).

Generation of single-strand DNA (ssDNA) gaps has been correlated with cell killing by PARPis [17]. To examine whether these previous findings were consistent with our results, we measured chromatin-bound replication protein A2 (RPA2), which reflects the amount of ssDNA in genomic DNA. Immunoblotting demonstrated that chromatin-bound RPA2 increased in TOV-112D SLFN11- KO/siBRCA2, parent/siCON, and furthermore increased in parent/siBRCA2 cells in the presence of 10 µM olaparib (Fig. 2A, B). Comparable results were obtained with niraparib (Supplementary Fig. S2). Thus, we conclude that chromatin-bound RPA2, which reflects an accumulation of ssDNA, was well correlated with impaired cell viability in response to PARPis (Fig. 1C, olaparib 10 µM). By contrast, under 100 nM CPT treatment, chromatin-bound RPA2 was increased similarly in SLFN11-KO/siCON and SLFN11-KO/siBRCA2 cells but was relatively restricted in parent/siCON and parent/siBRCA2 cells (Fig. 2C). Hence, chromatin-bound RPA2 was not correlated with cell killing in the case of CPT (Fig. 1B, CPT 100 nM). The results with CPT are consistent with our previous findings demonstrating that SLFN11 restricts the extension of uncoupled replication forks carrying ssDNA gaps [26]. The results of immunoblotting with olaparib treatment (Fig. 2A, B) matched well with the immunofluorescence with pre-extraction techniques that detect only chromatin-bound RPA2 (Fig. 2D, E). Together, these experiments demonstrate that PARPis generate ssDNA gaps that are enhanced by SLFN11 expression and BRCA2 deficiency, and even further in cells both proficient for SLFN11 and deficient for BRCA2.

### SLFN11 expression and BRCA1/2 deficiency increase ssDNA gaps in the presence of PARPis

**Fig. 3:**
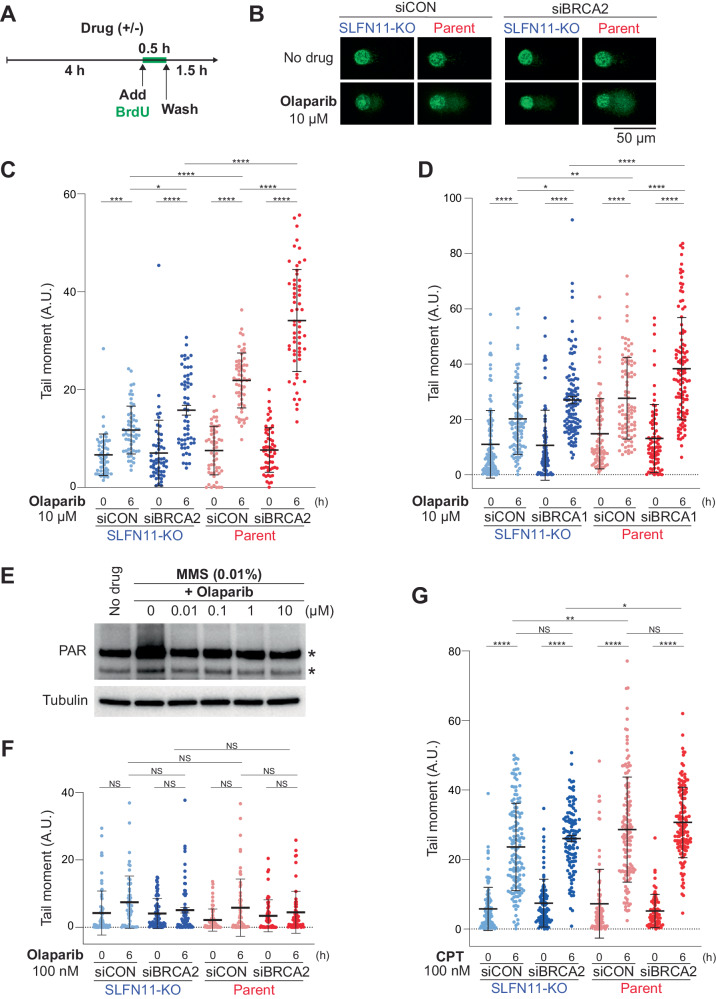
SLFN11 expression and BRCA1/2 deficiency increase ssDNA gaps induced by PARPis. **A** Scheme of the alkaline BrdU comet assay. **B** Representative alkaline BrdU comet assay images of TOV-112D cells treated with or without 10 μM olaparib. Scatter plots showing BrdU tail moments in TOV-112D cells under each drug treatment (**C**: 10 μM olaparib, **D**: 10 μM olaparib, **F**: 100 nM olaparib, **G**: 100 nM CPT). Data are means ± standard deviations (**C**: *n* = 54–59, **D**: *n* = 78–112, **F**: *n* = 54–77, **G**: *n* = 91–139, one-time experiment). **E** Immunoblots of PAR levels in whole cell lysates from parent TOV-112D cells treated as indicated for 30 min. Blots were probed with the indicated antibodies. The asterisk indicates a nonspecific band. NS not significant, *P < 0.05, **P < 0.01, ***P < 0.001, ****P < 0.0001 (one-way analysis of variance with Tukey’s post-hoc multiple comparisons test).

To further study the formation of ssDNA gaps induced by PARPis, we performed alkaline BrdU comet assays [18]. TOV-112D cells were treated with or without PARPis for 6 h, labeled with BrdU for 0.5 h during that time, and incubated in BrdU-free medium for the last 1.5 h (Fig. 3A). Tail moments of BrdU-labeled cells were measured to score ssDNA gaps [18]. Without drug treatment, tail moments were minimal and were not significantly affected by BRCA2 deficiency or SLFN11 expression (Fig. 3B, C). Olaparib (10 µM) increased the tail moments in SLFN11-KO/siCON cells and further increased in SLFN11-KO/siBRCA2 cells (Fig. 3B, C). Parent/siCON cells also exhibited significantly higher tail moments than SLFN11-KO/siCON cells, indicating that SLFN11 impairs OFP under olaparib treatment as BRCA2 deficiency does. Notably, parent/siBRCA2 cells exhibited the highest tail moments. Niraparib showed comparable results to olaparib (Supplementary Fig. S3A). The highest tail moments in parent/siBRCA2 compared to other conditions were verified in DAOY cell sets (Supplementary Fig. S3B). We also obtained comparable results among BRCA1-deficient and/or SLFN11-expressing conditions in TOV-112D cells (Fig. 3D). Together, these results demonstrate that SLFN11 expression increases ssDNA gaps in cells treated with PARPis and that this effect is independent of BRCA1/2 but enhanced in BRCA1/2-deficient cells.

We next examined whether catalytic inhibition or PARP-trapping was more important for OFP impairment. To this end, we first checked the catalytic inhibitory concentration for PARylation by olaparib. Because under normal conditions, endogenous PARylation was not detectable in TOV-112D cells, we used the alkylating agent methanesulfonate (MMS) to induce PARylation and titrate the olaparib concentration required for inhibiting PARylation (Fig. 3E) [6]. As olaparib suppressed PARylation even at 10 nM, we used 100 nM olaparib to maximally suppress PARylation. Under these conditions, tail moments were not increased under any conditions (Fig. 3F), suggesting that catalytic inhibition of PARP1/2 alone is insufficient to impair OFP. By contrast, 100 nM CPT increased tail moment but at similar levels in all cellular conditions (Fig. 3G), indicating that CPT induces ssDNA gaps regardless of SLFN11 expression and BRCA2 deficiency. According to the known mechanism of CPT action, these single-stranded DNA gaps are caused by the formation of TOP1-cleavage complexes (TOP1-trapping) [35]. Together, these results reveal the unique effects of PARPis in increasing ssDNA gaps in a SLFN11-dependent and BRCA1/2 deficiency manner likely through PARP-trapping at Okazaki fragments [16, 18].

### BRCA2 deficiency enhances the chromatin recruitment of SLFN11 in cells treated with PARPis

**Fig. 4:**
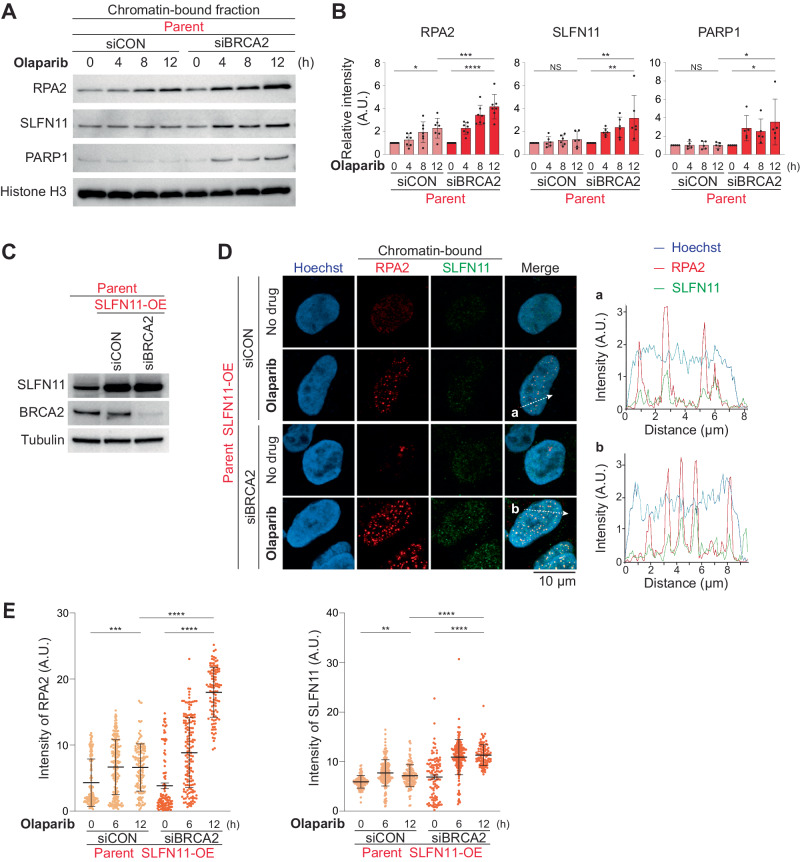
BRCA2 deficiency enhances the recruitment of SLFN11 on chromatin in PARPi-treated cells. **A** Representative immunoblots of chromatin-bound fractions prepared from TOV-112D cells treated with 10 μM olaparib for 0, 4, 8, or 12 h. Blots were probed with the indicated antibodies. **B** Quantification of data from **A**. Data were normalized to untreated cells (0 h). Data are means ± standard deviations (*n* = 5–7, biological replicates). **C** Immunoblots of whole cell lysates prepared from the indicated TOV-112D cells. Blots were probed with the indicated antibodies. **D** Representative confocal microscopy images; Hoechst (blue), chromatin-bound RPA2 (red), and SLFN11 (green) in TOV-112D cells. Cells were treated with or without 10 μM olaparib for 12 h. Representative tracings of the distribution of signals along the white dashed arrow (a and b) are shown in the merged panel. **E** Quantification of data from **D**. Scatter plots show mean signal intensities of RPA2 and SLFN11. Data are means ± standard deviations (*n* = 104–195, one-time experiment). NS not significant, *P < 0.05, **P < 0.01, ***P < 0.001, ****P < 0.0001 (one-way analysis of variance with Tukey’s post-hoc multiple comparisons test).

Given the ability of SLFN11 to bind RPA complex-coated ssDNA as well as ssDNA [25, 26] and our current finding that ssDNA gaps increase in SLFN11-expressing cells treated with PARPis, we hypothesized that SLFN11 could be recruited to the PARPi-induced ssDNA gaps. To test this possibility, we collected chromatin-bound fractions from TOV-112D parent/siCON or parent/siBRCA2 cells after olaparib treatment. Chromatin-bound PARP1 was increased in parent/siBRCA2 cells, indicating that more PARP-trapping lesions are generated under BRCA2-deficient conditions (Fig. 4A, B). While the chromatin recruitment of SLFN11 was not apparent in parent/siCON, the kinetics of the SLFN11 recruitment overall coincided with the kinetics of RPA2 signals in parent/siBRCA cells (Fig. 4A, B). These results demonstrate that SLFN11, RPA2 and PARP1 associate with chromatin with similar kinetics in cells treated with olaparib, especially in BRCA2-deficient conditions.

We next analyzed chromatin-bound SLFN11 using immunofluorescence with pre-extraction [26]. As the chromatin binding of endogenous SLFN11 was hard to detect in TOV-112D parent cells, we generated SLFN11-overexpressing (OE) TOV-112D parent cells (Fig. 4C). Knocking down *BRCA2* by siRNA was as effective as in the original parent cells (compare Figs. 1A and 4C). More importantly, in these parent/siBRCA2 cells treated with olaparib, chromatin-bound SLFN11 was increased to a greater extent than in the parent/siCON cells (Fig. 4D, E). In addition, line plots showed that the chromatin-bound SLFN11 was colocalized with chromatin-bound RPA2 (Fig. 4D). From these results, we conclude that SLFN11 binds RPA complex-coated ssDNA gaps under olaparib treatment and that BRCA2 deficiency generates more PARP-trapping and ssDNA gaps behind replication forks, where SLFN11 recruitment is enhanced.

### SLFN11 recruitment at ssDNA gaps behind replication forks does not block replication

**Fig. 5:**
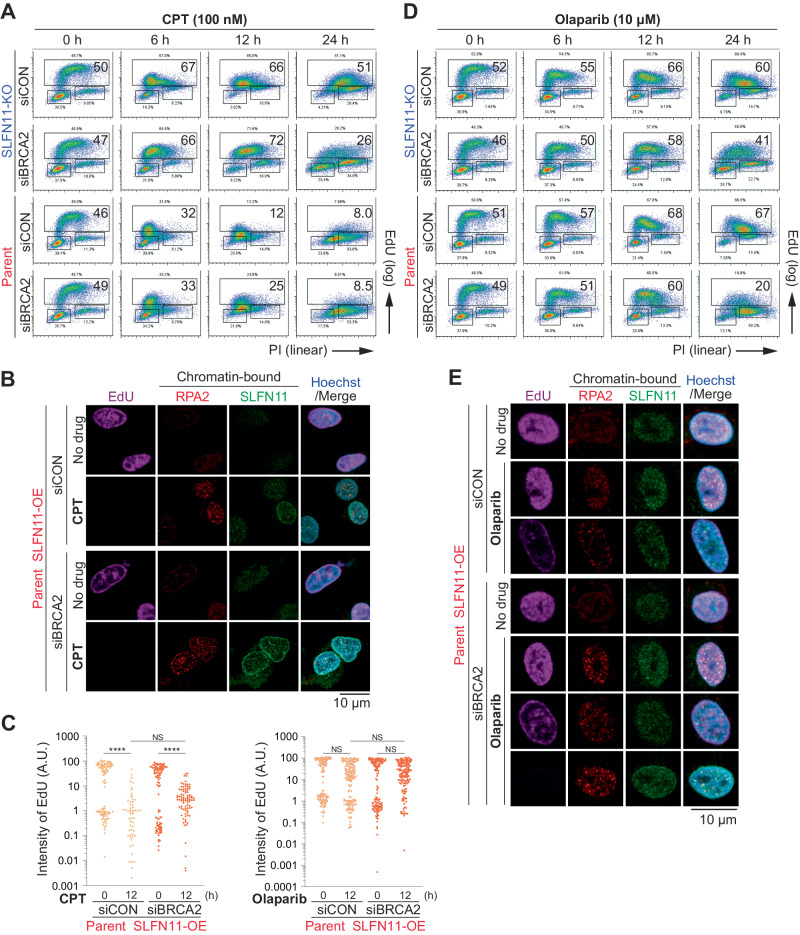
SLFN11 recruitment behind replication forks does not block replication. Representative flow cytometry cell cycle data in TOV-112D cells treated with 100 nM CPT (**A**) or 10 μM olaparib (**D**) for 6, 12, or 24 h. The percentage of highly replicating cells is annotated. Data represents one of two independent experiments. EdU 5-ethynil-2′-deoxyuridine, PI propidium iodide. **B**, **E** Representative confocal microscopy images; EdU (purple), Hoechst (blue), chromatin-bound RPA2 (red), and SLFN11 (green) in TOV-112D cells. Cells were treated with or without 100 nM CPT (**B**) and 10 μM olaparib (**E**) for 12 h. **C** Quantification of data from **B**, **E**. Scatter plots show the mean signal intensity of EdU. Data are means ± standard deviations (CPT: *n* = 58–102, olaparib: *n* = 126–164, one-time experiment). Data represents one of two independent experiments. NS not significant, ****P < 0.0001 (one-way analysis of variance with Tukey’s post-hoc multiple comparisons test).

To elucidate the potential differences between SLFN11 recruitment behind replication forks in response to PARP inhibition and SLFN11 recruitment at replication forks in response to CPT, we first performed cell cycle analyses. In response to CPT, SLFN11-KO/siCON cells resumed replication at 24 h (Fig. 5A, top) and SLFN11-KO/siBRCA2 cells showed a faster replication state than SLFN11-KO/siCON cells at 6, 12, and 24 h (Fig. 5A, second from top). This is likely because the S-phase checkpoint was less activated in SLFN11-KO/siBRCA2 cells than in SLFN11-KO/siCON cells, as inferred from a recent report [36] and the observed reduction of Checkpoint Kinase 1 (CHK1) phosphorylation at S345 (Supplementary Fig. S4A). SLFN11-expressing cells (parent/siCON and parent/siBRCA2 cells) treated with CPT showed a persistent replication block at 12 and 24 h (Fig. 5A, second from bottom and bottom). Yet, the S-phase checkpoint was less activated in BRCA2-deficient than in parent/siCON cells (Supplementary Fig. S4A), indicating the dominant replication-blocking effects of SLFN11 [26].

We next performed immunofluorescence for RPA2 and SLFN11 with pre-extraction in cells treated with 5-ethynyl-2’-deoxyuridine (EdU) for 1 h before harvesting to determine the location of replication foci. After CPT treatment for 12 h, SLFN11 formed foci at the nuclear periphery and the inner nucleus, where RPA2 colocalized (Fig. 5B, second from top and bottom) [26]. Cells with SLFN11 foci were mostly negative for EdU signals indicative of replication block in these cells and EdU-positive cells were significantly decreased (Fig. 5C, left). These results confirmed our previous findings that SLFN11 at replication forks blocks replication in response to CPT [26].

Parallel cell cycle analyses in TOV-112D cells treated with 10 µM olaparib showed a lesser impact on cell cycle progression at 6 h and 12 h compared to CPT in the four subsets. Additionally, cells in each subset kept progressing toward G2-phase until 24 h (Fig. 5D). BRCA2-deficient cells (SLFN11-KO/siBRCA2 and parent/siBRCA2) exhibited slightly faster cell cycle progression than each counterpart (SLFN11-KO/siCON and parent/siCON) at 24 h with less S-phase checkpoint activation (Fig. 5D and Supplementary Fig. S4B). These results indicate that, by contrast to CPT, SLFN11 has little impact on replication rate in cells treated with olaparib at concentrations that trap PARP1. Consistently, the EdU-positive population was not affected by olaparib (Fig. 5C, right) and chromatin-bound SLFN11 was mostly detected in EdU-positive cells with colocalization with RPA2 under olaparib treatment (Fig. 5E). The patterns of SLFN11 foci and RPA2 foci in the presence of olaparib were clearly different from those produced by CPT; where, as discussed above, the SLFN11 foci induced by CPT tended to be most visible at the nuclear periphery (Fig. 5B, E). Nevertheless, we detected a small number of cells with SLFN11 foci at the nuclear periphery and inner nucleus under olaparib treatment; however, these cells were negative for EdU overall (Fig. 5E, bottom). Comparable results were obtained with the DAOY cell set (Supplementary Figs. S4C-F, S5). Together, these results suggest that olaparib induces ssDNA gaps both behind replication forks and at replication forks, both of which recruit SLFN11 to chromatin, and the recruitment of SLFN11 behind replication forks does not block replication.

### Resection by MRE11 is required for SLFN11 recruitment to chromatin

**Fig. 6:**
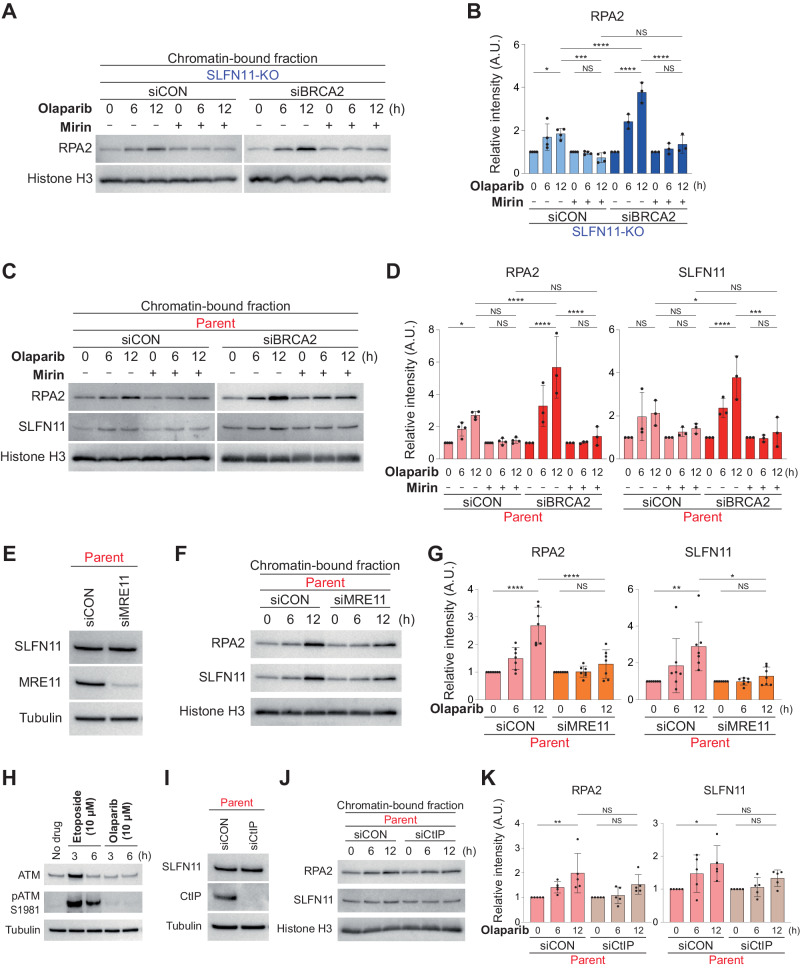
Resection by MRE11 is required for SLFN11 to be recruited on chromatin. **A** Immunoblots of chromatin-bound fractions prepared from TOV-112D (SLFN11-KO) cells treated with 10 μM olaparib and with or without 50 μM mirin for 0, 6, or 12 h. Blots were probed with the indicated antibodies. **B** Quantification of data from **A**. Data were normalized to untreated cells (0 h). Data are means ± standard deviations (*n* = 3–4, biological replicates). **C** Representative immunoblots of chromatin-bound fractions prepared from TOV-112D (parent) treated with 10 μM olaparib with or without 50 μM mirin for 0, 6, or 12 h. Blots were probed with the indicated antibodies. **D** Quantification of data from **C**. Data were normalized to untreated cells (0 h). Data are means ± standard deviations (*n* = 3–4, biological replicates). **E** Immunoblots of whole cell lysates prepared from the indicated TOV-112D cells: siCON or MRE11 siRNA (siMRE11). Blots were probed with the indicated antibodies. **F** Representative immunoblots of chromatin-bound fractions prepared from TOV-112D (parent) treated with 10 μM olaparib for 0, 6, or 12 h. Blots were probed with the indicated antibodies. **G** Quantification of data from **F**. Data were normalized to untreated cells (0 h). Data are means ± standard deviations (*n* = 7, biological replicates). **H** Representative immunoblots of whole cell lysates from parent TOV-112D cells treated with 10 μM etoposide or 10 μM olaparib for 0, 3, or 6 h. Blots were probed with the indicated antibodies. **I** Immunoblots of whole cell lysates prepared from the indicated TOV-112D cells: siCON or CtIP siRNA (siCtIP). Blots were probed with the indicated antibodies. **J** Representative immunoblots of chromatin-bound fractions prepared from TOV-112D (parent) treated with 10 μM olaparib for 0, 6, or 12 h. Blots were probed with the indicated antibodies. **K** Quantification of data from **J**. Data were normalized to untreated cells (0 h). Data are means ± standard deviations (*n* = 5, biological replicates). NS not significant, *P < 0.05, **P < 0.01, ***P < 0.001, ****P < 0.0001 (one-way analysis of variance with Tukey’s post-hoc multiple comparisons test).

Given the accumulation of ssDNA gaps in SLFN11-expressing cells lacking BRCA1/2, we tested the role of the 3’ to 5’ meiotic recombination 11 (MRE11) exonuclease in the generation of the ssDNA gaps observed under olaparib treatment. We treated the four subsets of TOV-112D cells with olaparib and with or without mirin, an established MRE11 inhibitor. In SLFN11-KO/siCON and SLFN11-KO/siBRCA2 cells in which olaparib treatment increased chromatin-bound RPA2, the addition of mirin decreased the chromatin-bound RPA2 almost to control levels (Fig. 6A, B). In the SLFN11-expressing cells (parent/siCON and parent/siBRCA2) treated with olaparib, chromatin-bound RPA2 and SLFN11 were also decreased to control levels by the addition of mirin (Fig. 6C, D). These results were further corroborated using siRNA targeting MRE11 (Fig. 6E–G). Thus, MRE11-mediated resection is likely critical for generating ssDNA gaps in response to PARPis, regardless of BRCA2 or SLFN11.

Upon the occurrence of DNA double-strand breaks (DSBs), the Mre11/Rad50/NBS1 (MRN) nuclease complex maintains genomic stability by bridging DNA ends and initiating DNA damage signaling by activating the ataxia-telangiectasia mutated (ATM) kinase [37]. To assess the necessity of ATM activation during ssDNA gap formation by MRE11 in the context of olaparib treatment, we evaluated ATM phosphorylation at S1981—a recognized marker for ATM activation. As a positive control, we used the topoisomerase II (TOP2) poison etoposide, known to induce TOP2-mediated DSBs. While etoposide-induced phospho-ATM, olaparib did not (Fig. 6H). This suggests that MRE11, or the MRN complex, operates independently of ATM activation in response to olaparib. Downregulation of the C-terminal-binding protein (CtBP)-interacting protein (CtIP), which collaborates with the MRN complex in DNA end resection [38], also diminished the chromatin recruitment of both RPA2 and SLFN11 (Fig. 6I–K). These findings indicate that the MRN complex, in conjunction with CtIP rather than MRE11 alone, is responsible for generating ssDNA gaps in response to PARP inhibitors without triggering ATM activation.

### SLFN11 expression is a biomarker of favorable response to olaparib in ovarian cancers

**Fig. 7:**
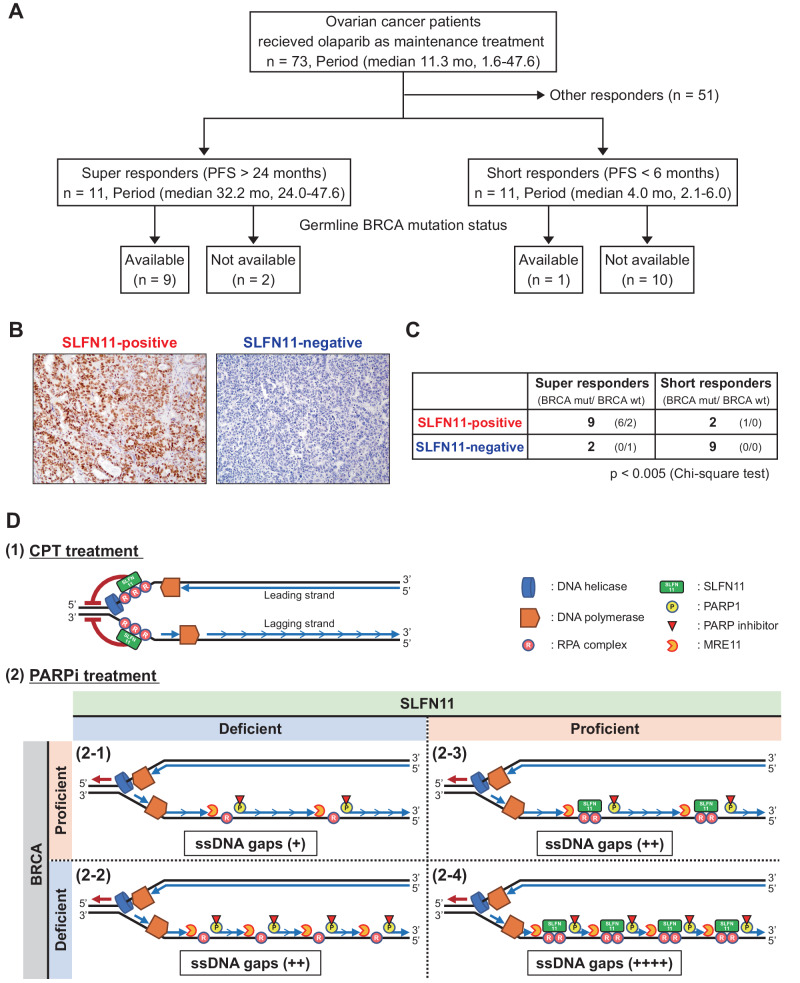
SLFN11-positive ovarian cancers exhibit better responses to olaparib. **A** Participant flow. **B** Representative immunohistochemical images of SLFN11-positive and SLFN11-negative ovarian cancer samples (magnification ×400). **C** The chart depicts SLFN11 expression level in two groups: super-responders and short-responders (*N* = 11 each). *P* < 0.005 (chi-square test). Information on BRCA status is provided in parentheses. **D** Proposed model describing the involvement of SLFN11 and BRCA for CPT (as a typical DNA damaging agent) vs. PARPi treatment. Please see the Discussion section for details.

Given that SLFN11 expression, both alone and in combination with BRCA deficiency, leads to increased ssDNA gaps and hypersensitivity to PARPis, we conducted a retrospective analysis of SLFN11 expression using immunohistochemistry in a cohort of 73 ovarian cancer patients who had received olaparib as maintenance therapy at Keio University Hospital (Fig. 7A, B). The median duration of olaparib treatment was 11.3 months, with a range of 1.6–47.6 months. In our examination of progression-free survival (PFS), we categorized patients into two groups: super-responders, defined as having more than 2 years without disease progression under olaparib treatment, and short-responders, who experienced disease progression within 6 months during olaparib treatment. A significantly higher number of SLFN11-positive cases was observed among the 11 super-responders (median 32.2 months; 24.0–47.6 months) compared to the 11 short-responders (median 4.0 months; 2.1–6.0 months) (Fig. 7C). Germline BRCA status was available for 9 out of the 11 super-responders, and 6 out of those 9 patients had BRCA mutations. The 6 patients with BRCA mutation were all positive for SLFN11 (Fig. 7C). Although the analysis was based on a limited number of samples as responses to PARP inhibitors beyond two years are rare, these findings suggest that SLFN11 expression may serve as a predictive biomarker for identifying patients who are likely to respond favorably to olaparib maintenance therapy.

## Discussion

Accumulation of ssDNA gaps behind replication forks has recently emerged as a mechanism for synthetic lethality in BRCA-deficient cells treated with PARPis [17]. In this study, we show that SLFN11 is recruited to the ssDNA generated by PARPis and that it increases those gaps (Figs. 2–5). To the best of our knowledge, this is the first study to identify an enhancing factor of ssDNA gaps through binding to such gaps. We also demonstrate that the PARPi-induced ssDNA gaps are further enhanced in SLFN11-proficient cells with BRCA1/2 deficiency. This finding explains at least in part why SLFN11 enhances the antiproliferative activity of PARPis in BRCA1/2-deficient cancer cells (Fig. 1). We find that inhibition of PARP1/2 catalytic activity is insufficient to generate the ssDNA gaps (Fig. 3), suggesting that PARP-trapping is the primary mechanism for inducing the ssDNA gaps.

Although PARP-trapping, demonstrated by increased PARP1 in the chromatin fraction, was only obvious under BRCA2-deficient conditions (Fig. 4A), we assume that undetectably low levels of PARP-trapping also occur under BRCA2-proficient conditions. Moreover, the increased binding of RPA2 on chromatin was reduced by MRE11 inactivation under all conditions (Fig. 6A–G) indicating that resection by MRE11 is a critical step for generating the ssDNA gaps. Given that CtIP plays a role in ssDNA gap formation (Fig. 6I–K), we speculate that the MRN complex with CtIP rather than MRE11 alone generates the ssDNA gaps. Although olaparib did not induce ATM activation (Fig. 6H), potential ATR activation (Supplementary Fig. S4B, F) may activate CtIP [39].

Based on these results, we propose the working model illustrated in Fig. 7D. The lagging strand comprises a series of Okazaki fragments, approximately 200 nucleotides (nt) in length, where the junctions become indistinguishable post-ligation (Fig. 6D, depicted by sequential arrows). In the case of CPT (Fig. 7D.1) (or a typical DNA damaging agent) inducing replication stress associated with the uncoupling of the replication helicase and polymerases, RPA-coated ssDNA gaps form at replication forks, and SLFN11 binds to those gaps and blocks replication [26] (Fig. 5). In the case of PARP inhibitors, ssDNA gaps are generated behind forks; yet the length and the number of ssDNA gaps have not been documented and they could vary among conditions. Given that approximately millions of Okazaki fragments are generated during one cell cycle and that DNA replication speed is 100 nt/sec, Okazaki fragments are ligated every 2 seconds. Hence, we hypothesize that in the presence of PARP inhibitors ssDNA gap form at Okazaki fragments in response to PARP-trapping (Fig. 7D.2). In SLFN11-deficient/BRCA-proficient cells (Fig. 7D.2-1), PARP-trapping impairs OFP, generating DNA ends, followed by MRE11-dependent end-resection (see Fig. 6A, B). However, the ssDNA gap length or the number of resection sites remains limited (see Figs. 2 and 3). Hence, PARPi sensitivity is also limited (Fig. 1). In SLFN11-deficient/BRCA-deficient cells (Fig. 7D.2-2), dual inhibitory effects of OFP by BRCA deficiency and PARP-trapping generate more DNA ends, followed by the MRE11-dependent resection, resulting in ssDNA gap accumulation. Moreover, the defective S-phase checkpoint associated with BRCA2 deficiency (Supplementary Fig. S4) may provide more Okazaki fragments within a certain time point and contribute to ssDNA gap accumulation. Figure 7D.2-3 proposes that SLFN11 is recruited to the ssDNA gaps after resection by MRE11 (Fig. 6C, D). The mechanisms through which SLFN11 increases ssDNA gaps were not examined in this study and warrant further investigations. It is plausible that chromatin-bound SLFN11 physically or enzymatically blocks the function of unknown polymerases or primases that fill the ssDNA gaps. Otherwise, SLFN11 on chromatin somehow stabilizes the ssDNA gaps. BRCA deficiency causes more ssDNA gaps (Fig. 7D.2-4), leading to enhanced SLFN11 recruitment. Consequently, PARPis cause the increased accumulation of ssDNA gaps in BRCA-deficient/ SLFN11-proficient cells and produce the highest cell-killing effect (Fig. 1). In summary, we propose that SLFN11 at replication forks blocks replication, whereas SLFN11 behind replication forks does not affect replication but increases toxic ssDNA gaps. In addition to the previously known functions of SLFN11 in replication stress [40–46], our study demonstrates that SLFN11 also enhances the accumulation of ssDNA gaps behind replication forks.

### Dual modes of action of PARPis: PARP-trapping at Okazaki fragments or at intermediates of BER

We previously showed that PARPis trap PARP1 and PARP2 at 5’-ssDNA ends of base excision repair (BER) intermediates arising from endogenous reactive oxidative stress and exogenous base damages [47]. The encounter between those PARP-trapping lesions and ongoing replicons results in DSBs that kill cancer cells. Talazoparib is the strongest PARP-trapping PARP inhibitor [6–8, 48] that could act in a manner similar to CPT, which forms TOP1 cleavage complexes that block replication progression (Fig. 7D.1). Indeed, we have demonstrated replication block by SLFN11 under talazoparib single treatment [9]. With talazoparib, PARP-trapping is also likely to occur behind the replication forks at Okazaki fragments; however, the replication blocks will suppress the generation of new Okazaki fragments. Thus, the replication block induced by SLFN11 may counteract the accumulation of ssDNA gaps. Because a relatively low concentration of talazoparib exhibited synergistic effects in SLFN11-proficient cells with BRCA2 deficiency (Fig. 1), the dual modes of PARP-trapping may determine how cells die. Indeed, the weakest PARP-trapping PARP inhibitor veliparib showed a synergistic effect even at 25 µM (Fig. 1). As our findings demonstrated, 10 µM olaparib exhibited dual effects, but the induction of ssDNA gaps behind replication forks was dominant (Fig. 5). Because the Cmax (maximum concentration in human serum) of olaparib is approximately 20 µM, we summon that a dominant mechanism of action for olaparib in the clinical setting is the generation of ssDNA gaps behind replication forks.

### Translational clinical implications

Our study unveils the significance of SLFN11 as a beneficial factor for an extended response to olaparib in clinical settings (Fig. 7C). Although the limited sample size restricts further analysis of the contribution of BRCA status, extensive research has already demonstrated the positive impact of BRCA mutations in enhancing olaparib response. Therefore, our findings provide novel mechanistic insights into potential favorable responses to olaparib in ovarian cancers characterized by high SLFN11 expression and BRCA-inactivating mutations. These observations underscore the importance of assessing SLFN11 expression, in addition to the BRCA status in clinical practice [49], to identify optimal responders to olaparib. The implication of SLFN11 for PARPis is different from that of general DNA-damaging agents (Fig. 7D). This notion is important for interpreting clinical data and biomarkers in clinical trials.

## Materials and methods

### Cell culture and drug treatments

TOV-112D (RRID: CVCL_3612) and DAOY (RRID: CVCL_1167) cells were purchased from American Type Culture Collection (ATCC; Manassas, VA, USA). Cell line authentication was obtained by short tandem repeat analysis (Promega, USA). Cells were tested negative for mycoplasma contamination. TOV-112D and DAOY cells were grown in Dulbecco’s modified Eagle medium (cat. no. 044-29765; Wako, Japan) with 10% fetal bovine serum (cat. no. 536-90165; Hyclone, Cytiva), 20 U/mL penicillin (Meiji-Seika Pharm, Japan), and 100 μg/mL streptomycin (Meiji-Seika Pharma) at 37 °C in 5% CO_2_. Camptothecin (CPT) was obtained from Cayman Chemical Company (cat. no. 11694), talazoparib was from MedChemExpress (cat. no. HY-166106), olaparib was from LC Laboratories (cat. no. O-920), and MMS was from Tokyo Chemical Industry (cat. no. M0369). Niraparib (cat. no. S2741), veliparib (cat. no. S1004), and mirin (cat. no. S8096) were obtained from Selleckchem.

### Generation of *SLFN11*-deleted cells

SLFN11-knockout (KO) cells in DAOY were previously published [32] and SLFN11-KO cells in TOV-112D were generated in another study [33]. Disruption of the *SLFN11* gene using the CRISPR/Cas9 method was reported previously [9]. Briefly, each guide RNA (5′-gcgttccatggactcaagag-3′ or 5′-gttgagcatcccgtggagat-3′) was inserted into the pX330 plasmid (pX330-SLFN11, RRID: Addgene_101733). Gene-targeting constructs harboring homology arms and a puromycin-resistance cassette were prepared. The targeting construct and pX330-SLFN11 were co-transfected into TOV-112D and DAOY cells by lipofection. After transfection, cells were released into drug-free medium for 48 h followed by puromycin selection until single colonies were formed. Single clones were expanded, and gene-deletion was confirmed by western blotting. This study was approved by Genetic Modification Commission in Ehime University and Keio University and carried out according to the guidelines of the committee.

### Generation of *SLFN11*-overexpressing cells

pPCIP-cSLFN11 was first made by insertion of Xho I-digested 5’- and 3’-terminal inverted repeats cassette sequences of the piggyBac system [50] amplified by PCR using two oligos: 5’– CCGCTCGAGTTAACCCTAGAAAGATAATCATATTGTGACGTACGTTAAAGATAATCATGCGTAAAATTGACGCATGTTCGAAATGCATGG and 5’-CCGCTCGAGTTAACCCTAGAAAGATAGTCTGCGTAAAATTGACGCATGCGAATTCGGTACCATGCATTTCGAACATGCG into the Sal I-digested pcDNA3 vector (RRID: Addgene_15475) [51] to create the pcDNA-IRs. The CAG promoter digested with Mph1103 I and Acc65 I from pCAG-BSD vector (WAKO) cloned into pcDNA-IRs with Mph1103 I and Acc65 I to create pPC. The 3xFL-IRES-PuroR-HSV TK poly (A) signal fragment was PCR amplified from pMX-3xFL-IP [52] using primers 5’-CGGAATTCATGGGCGTTGCCATGCCAGGTGCCGAAGATGATGTGGTGTAACAATTCATGGACTACAAAGACCATGACGG and 5’-ACATGCATGCGAACAAACGACCCAACACCGTGCGTTTTATTCTGTCTTTTTATTGCCGGTCGACTCAGGCACCGGGCTTGCGGG. The PCR product was cloned into pPC with EcoR I and Pae I to create pPCIP. The full length of SLFN11 cDNA was amplified using primers 5’-

AGATCTGGATCCAGCATGGAGGCAAATCAGTGCC and 5’-

CACGCGTCTCGAGGCCTAATGGCCACCCCACGGAA, and integrated into the NotI site of the pPCIP vector, creating pPCIP-cSLFN11 vector, using Thermo GeneArt Seamless Cloning and Assembly Enzyme Mix (cat. no. A14606; Thermo Scientific). The final products were validated by sequencing.

The expression vector containing hyperactive PB transposase cDNA under CAG promoter (pCAG2-hyPB) was used before [53]. Further plasmid information is available from the Mendeley link: 10.17632/cr443kztj9.1.

An SLFN11 expression vector (pPCIP-cSLFN11) and the expression vector of a hyperactive PB transposase (pCAG2-hyPB) were co-transfected into TOV-112D and DAOY cells by lipofection using Lipofectamine 3000 Reagent (cat. no. L30000-015; Invitrogen, Carlsbad, CA, USA), according to the manufacturer’s instructions. Four days after transfection, cells were incubated in medium containing puromycin (0.2 μg/mL) for another 10 days, and surviving cells were used for the assays.

### Small interfering RNA (siRNA) transfection

Gene-specific siRNAs (mix of 4 sequences) for human BRCA1 (cat. no. L-003461-00-0005), human BRCA2 (cat. no. L-003462-00-0010), human MRE11 (cat. no. L-009271-00-0005), human CtIP (cat. no. L-011376-00-0005) and negative control siRNAs (cat. no. D-001810-10-15) were products of Dharmacon. According to the manufacturer’s instructions, each siRNA (10 nM) was transfected into cells using Lipofectamine RNAimax Reagent (cat. no. 13778-150; Invitrogen). The culture medium was changed 6 h after the transfection, and subsequent experiments were performed 24 h after transfection.

### Antibodies

Antibodies against SLFN11 (cat. no. sc-515071) and CHK1 (cat. no. sc-8408, RRID: AB_627257) were obtained from Santa Cruz Biotechnology (Santa Cruz, CA, USA); antibodies against BRCA1 (cat. no. 9010, RRID: AB_2228244), BRCA2 (cat. no. 10741, RRID: AB_2797730), RPA2 (cat. no. 35869, RRID: AB_2799086), histone H3 (cat. no. 4499, RRID: AB_10544537), PAR (cat. no. 83732, RRID: AB_2749858), PARP1 (cat. no. 9532, RRID: AB_2749858), phospho-CHK1 (S345) (cat. no. 2348, RRID: AB_331212), ATM (cat. no. 2873, RRID: AB_2062659), phospho-ATM (S1981) (cat. no. 4256, RRID: AB_2062663), MRE11 (cat. no. 4895, RRID: AB_2145100), CtIP (cat. no. 9201, RRID: AB_10828593) were from Cell Signaling Technology (Danvers, MA, USA); and antibodies against α-tubulin (cat. no. 017-25031) were from Wako. Human SLFN11 monoclonal antibodies (cat. no. 5-14.12; mAbProtein Co., Ltd., Japan) were used for immunoblotting.

### Immunoblotting and quantification

To prepare whole cell lysates, cells were lysed with Laemmli SDS sample buffer with sonication and boiling. To prepare chromatin-bound subcellular fractions, we followed the protocol for a Subcellular Protein Fractionation Kit for Cultured Cells (cat. no. 78840; Thermo Scientific). Protein concentration in cell lysates was measured using an RC DC Protein Assay Kit (cat. no. 50000119, RRID: SCR_008426; Bio-Rad Laboratories, Hercules, CA, USA). Immunoblotting was carried out using standard procedures. Secondary antibodies were horseradish peroxidase-conjugated anti-mouse antibodies (cat. no. W402B, RRID: AB_430834; Promega, Madison, WI, USA) or rabbit IgG (cat. no. W401B, RRID: AB_430833; Promega). Protein signals were visualized using an iBright CL1500 Imaging system (Invitrogen). Band intensity was quantified using ImageJ software (RRID: SCR_003070).

### Cell viability assay

TOV-112D cells (1.5 × 10^3^) and DAOY cells (1.0 × 10^3^) were seeded into 96-well white plates (cat. no. SPL-30196; SPL Life Sciences) in 100 μL medium per well. Cells were continuously exposed to the indicated drug concentrations for 48 h in triplicate. Cellular viability was determined using an ATPlite 1step Kit (cat. no. 6016731; Perkin Elmer). Briefly, 25 μL ATPlite solution was added to each well of a 96-well plate. After 5 min, luminescence was measured with a FlexStation3 (Molecular Devices) or Varioskan LUX multimode microplate reader (Thermo Scientific). The ATP level in untreated cells was defined as 100%. The viability (%) of treated cells was defined as ATP in treated cells/ATP in untreated cells × 100.

### Apoptosis analysis by flow cytometry

We used an Annexin V-FITC Apoptosis Detection Kit (cat. no. 15342-54, Nacalai Tesque) to detect cell apoptosis, according to the manufacturer’s instructions. Data were collected with a Gallios Flow Cytometer (RRID: SCR_019639; Beckman Coulter) and analyzed with FlowJo software (RRID: SCR_008520; Becton Dickinson).

### Immunofluorescence analysis with or without Edu labeling

Cells were seeded on chamber slides (cat. no. 192-004; Watson). If needed, cells were incubated with 10 μM 5-ethynyl-2’-deoxyuridine (EdU) for 1 h before being collected. The cells were pretreated with cold 0.2% TritonX-100/phosphate-buffered saline (PBS) on ice for 1 min. The cells were then fixed with 4% paraformaldehyde in PBS for 15 min and incubated with 3% bovine serum albumin (BSA)/PBS for 10 min. Next, when EdU detection was necessary, cells were incubated with Click-iT Plus reaction cocktail for 30 min (cat. no. C10640; Invitrogen). Cells were incubated overnight with primary antibodies in 3% BSA/PBS at 4 °C. After washing with TBST, the cells were incubated with proper secondary antibodies and Hoechst33342 (cat. no. H3570; Invitrogen) in 3% BSA/TBST for 1 h. Anti-mouse IgG Alexa Fluor 488 (cat. no. A11001, RRID: AB_2534069; Invitrogen) or anti-rabbit IgG Cyanine3 (cat. no. A10520, RRID: AB_2534029; Invitrogen) were used as secondary antibodies. After washing with TBST, cells were mounted with ProLong Gold Antifade Mountant (cat. no. P36930; Invitrogen). Images were captured with a Nikon ECLIPSE Ti (RRID: SCR_021242) or ZEISS LSM 900 (RRID: SCR_022263) confocal microscope.

### Data analysis of immunofluorescence microscopy images

The signal intensity in each cell was measured using ImageJ software. A proper-sized circle was set slightly larger than a regular cell’s size. The same circle was used to measure the mean intensity of each signal throughout the experiment in isogenic cell lines. The individual signals were plotted. The line plot was obtained using ZEN software. The data were transferred to GraphPad Prism 7 software (RRID: SCR_002798) and illustrated.

### Alkaline BrdU comet assay

We overall followed the previous publication [54]. To detect ssDNA gaps, cells were pulse-labeled with 100 µM bromodeoxyuridine (BrdU) (cat. no. 05650-95; Nacalai Tesque) for 1 h, followed by incubation for 90 min as the chase period. We used a Comet Assay Single Cell Gel Electrophoresis Assay kit (cat. no. 4250-050-K; R&D Systems, Bio-Techne) according to the manufacturer’s instructions. Cells (1 × 10^5^/mL) were combined with molten LM Agarose (at 37 °C) at a ratio of 1:10 (v/v) and immediately pipetted onto CometSlides. Plated slides were incubated on a flat surface at 4 °C in the dark for 10 min, immersed in 4 °C lysis solution for 30 min, and immersed in Alkaline Unwinding Solution for 20 min. Electrophoresis was conducted at 1 V/cm (25 V, 300 mA) for 20 min at 4 °C in Alkaline Electrophoresis Solution. Excess electrophoresis solution was drained off, and slides were washed three times for 5 min each by layering with neutralization buffer (0.4 M Tris-HCl, pH 7.4). Slides were then immersed in 70% ethanol for 5 min, dried at 37 °C for 10 min, washed three times for 10 min each in PBS, and blocked with 0.1% Tween20/3% BSA/PBS for 30 min by horizontal layering at room temperature. After blocking, slides were incubated with the mouse monoclonal anti-BrdU antibodies (cat. no. 347580, RRID: AB_400326; Beckton Dickinson; 1:250) overnight at 4 °C in a humid chamber, then washed three times for 10 min each in TBST and incubated with secondary antibodies (anti-mouse Alexa Fluor 488, cat. no. A11001, RRID: AB_2534069; Invitrogen; 1:250)/0.1% Tween20/3% BSA/PBS for 1 h at room temperature in the dark. Thereafter, slides were washed three times for 10 min in TBST, covered with coverslips, viewed slides with a Zeiss Axio Observer Z1, and scored with CaspLab software (RRID: SCR_007249).

### Cell cycle analysis by flow cytometry

Cells were incubated with 10 μM EdU for 1 h using a Click-iT EdU Alexa Fluor 647 Flow Cytometry Assay Kit (cat. no. C10419; Invitrogen) according to the manufacturer’s instructions. Propidium iodide (cat. no. P4170; Sigma-Aldrich, St. Louis, MO, USA) was used to measure DNA content. Data were collected with a Gallios Flow Cytometer and analyzed with FlowJo software.

### Patients and clinical tissue samples

Retrospective clinical information was collected from medical records, including patient characteristics, germline BRCA1/2 status, and clinical response to olaparib maintenance therapy. Sample tissues were obtained from 73 patients with ovarian cancer who underwent surgery at Keio University Hospital (Tokyo, Japan) from 2018 to 2023. All specimens were fixed in 10% phosphate-buffered formalin and embedded in paraffin. The 4-μm-thick sections were stained with hematoxylin and eosin to confirm the presence of tumor and to assess its biological characteristics.

### Ethics approval and consent to participate

The human subjects, human materials, and human data in this study are in accordance with the Declaration of Helsinki. Written informed consent was obtained from each participant regarding the use of samples for research. The Ethics Committee of Keio University approved this study (approval No. 20070081).

### Immunohistochemical staining for SLFN11 and evaluation

The specimens were deparaffinized in xylene and rehydrated in a graded series of ethanol. Antigen retrieval was performed with citrate buffer (pH 6.0) for 20 min at 95 °C. After blocking endogenous peroxidase activity with 0.3% H_2_O_2_ in phosphate-buffered saline, the specimens were incubated overnight with mouse anti-SLFN11 antibody (cat. no. sc-515071) (1:500 dilution) at 4 °C. Indirect immunohistochemical staining was performed using N-Histofine Simple Stain MAX-PO (Nichirei Biosciences Inc.). The specimens were counterstained with hematoxylin, dehydrated in a graded series of ethanol, dried and coverslipped. All staining was manually scored blindly by one gynecologist (Y.K.) without knowledge of clinical and patient outcomes. SLFN11 positive was defined as >10% of tumor cells showing higher immunoreactivity compared to their stromal counterparts in 5 high-power fields in each section.

### Evaluation of germline BRCA status

Germline BRCA1/2 status was analyzed by BRACAnalysis® (Myriad Genetic Laboratories, Inc.).

### Statistical analysis

Statistical analyses were carried out using GraphPad Prism 7 software and conducted using one-way analysis of variance with Tukey’s post-hoc multiple comparisons test. Relevant comparisons are shown (NS: not significant, *P < 0.05, **P < 0.01, ***P < 0.001, ****P < 0.0001). Graphs show mean ± standard deviations (SD).

### Supplementary information

Supplemental Figures and legends S1-S5

## Data Availability

The unprocessed and uncompressed imaging data and the proof of cell line authentication are accessible at the following Mendeley link: 10.17632/cr443kztj9.1
