# Supplementary material for: Schlafen 11 further sensitizes BRCA-deficient cells to PARP inhibitors through single-strand DNA gap accumulation behind replication forks
Source: Oncogene. 2024 Jul 3;43(32):2475–89. doi: 10.1038/s41388-024-03094-1 (PMC11315672; doi:10.1038/s41388-024-03094-1)
Supplement: Supplementary file 1 — Supplemental Figures and legends S1-S5 [file 41388_2024_3094_MOESM1_ESM.pdf]

Figure S1

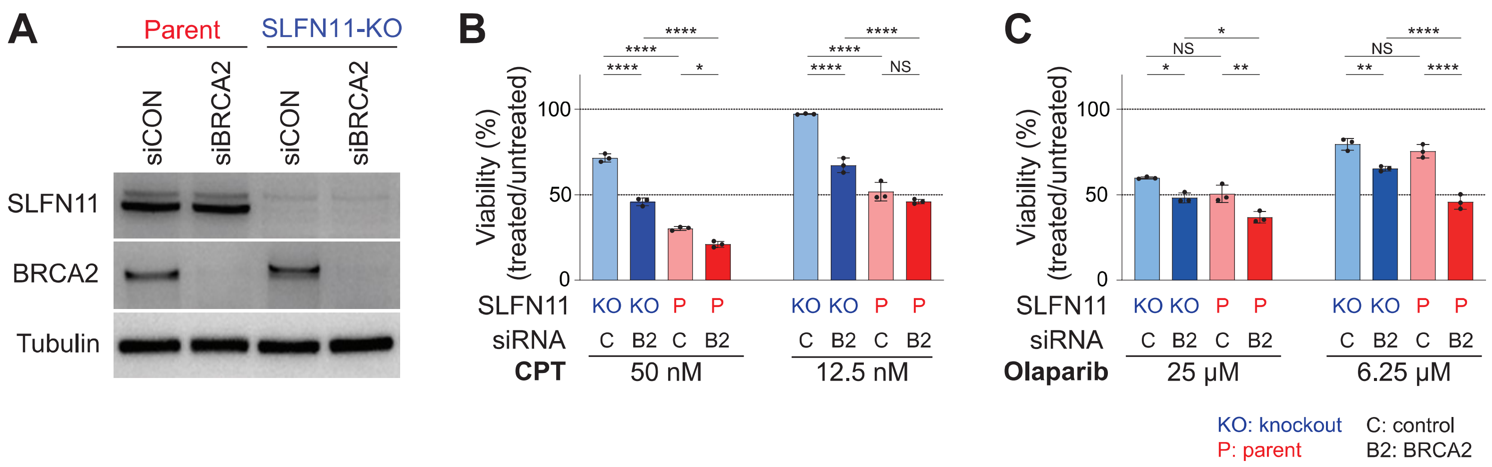

Figure S1. SLFN11 enhances cellular sensitivity to olaparib in BRCA2-deficient cells. **A**, Immunoblots of whole cell lysates from genetically modified DAOY cells: SLFN11-proficient (parent), SLFN11-KO, control siRNA (siCON) or BRCA2 siRNA (siBRCA2). Blots were probed with the indicated antibodies. **B-C**, Viability of DAOY cells under each condition after 48 h of continuous drug treatment. Cellular ATP activity was used to measure cell viability. The viability of untreated cells was set as 100%. Data are means ± standard deviations (n = 3, biological replicates). NS: not significant, **P* < 0.05, ***P* < 0.01, *****P* < 0.0001 (one-way analysis of variance with Tukey's post-hoc multiple comparisons test).

Figure S2

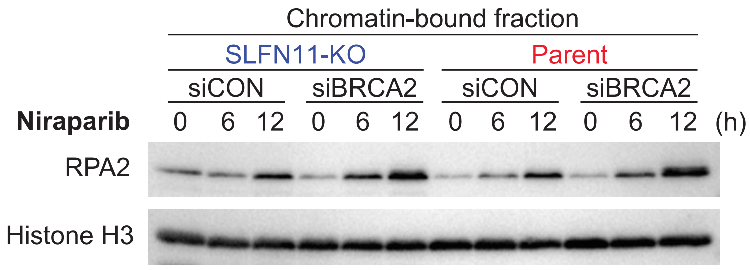

Figure S2. SLFN11 expression and BRCA2 deficiency increase chromatin-bound RPA2 under PARPi treatments. Representative immunoblots of chromatin-bound fractions prepared from the indicated TOV-112D cells treated with 1.2 μM niraparib for 0, 6, or 12 h. Blots were probed with the indicated antibodies.

Figure S3

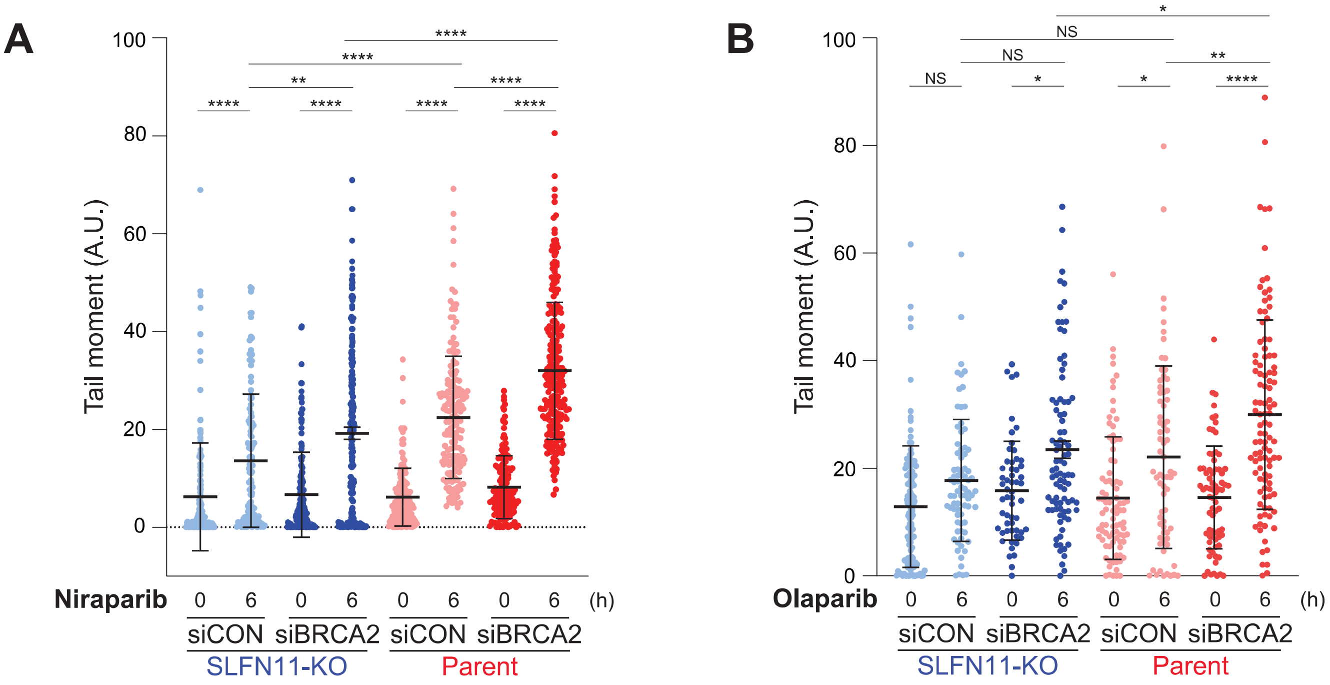

Figure S3. SLFN11 expression and BRCA1/2 deficiency increase ssDNA gaps induced by PARPis.

A, Scatter plots showing BrdU tail moments in TOV-112D cells treated with or without 1.2 µM niraparib. Data are means ± standard deviations (n = 119–241, one-time experiment). ***P* < 0.01, *****P* < 0.0001 (one-way analysis of variance with Tukey's post-hoc multiple comparisons test).

B, Scatter plots showing BrdU tail moments in DAOY cells treated with or without 10 µM olaparib Data are means ± standard deviations (n = 56–121, one-time experiment). NS: not significant, **P* < 0.05, ***P* < 0.01, *****P* < 0.0001 (one-way analysis of variance with Tukey's post-hoc multiple comparisons test).

Figure S4

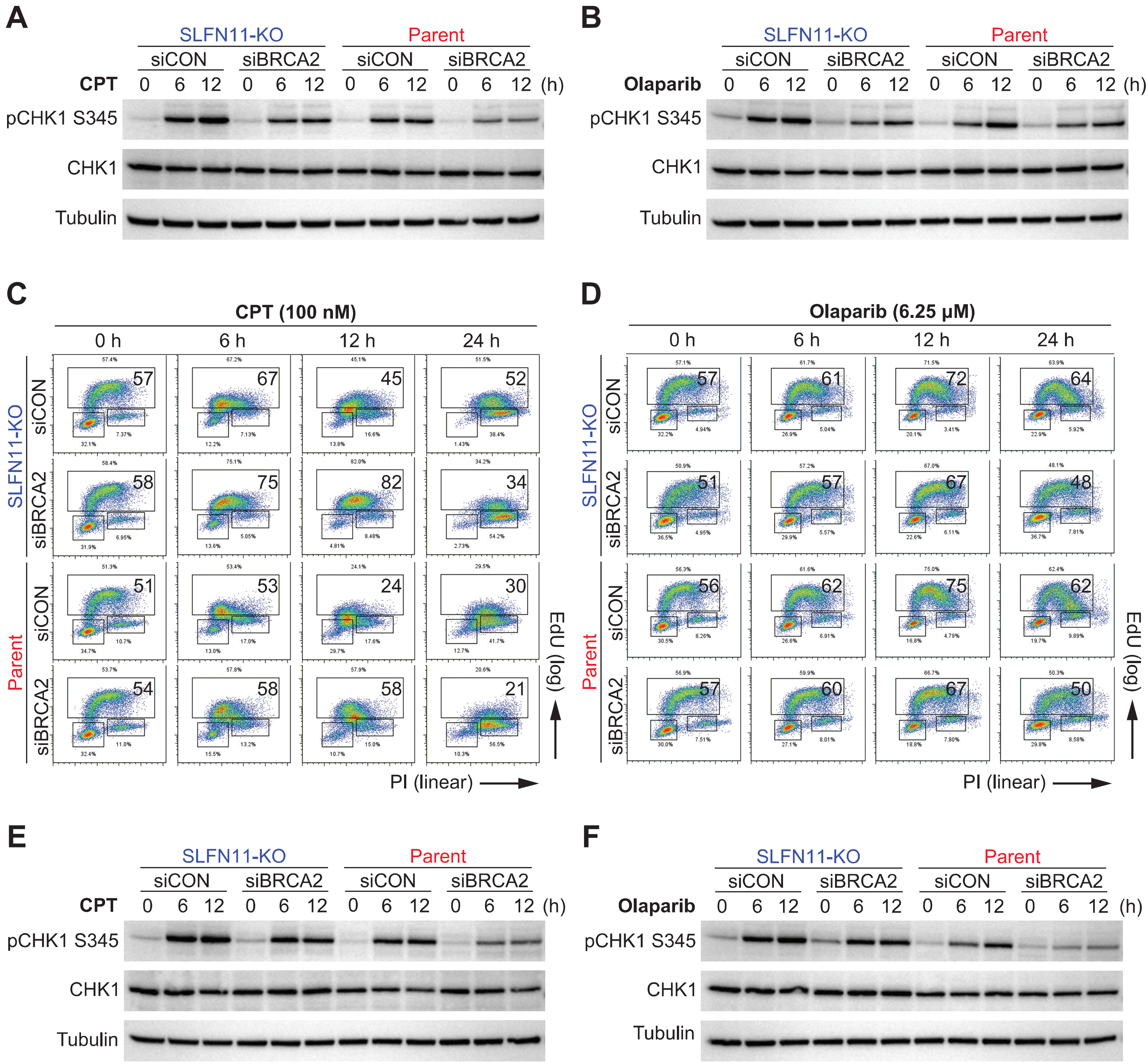

Figure S4. The S-phase checkpoint is less activated in BRCA2-deficient cells than in the BRCA2-proficient cells. **A**, **B**, Representative immunoblots of whole cell lysates from the indicated TOV-112D cells treated with 100 nM CPT (**A**) and 10 µM olaparib (**B**). Blots were probed with the indicated antibodies. **C**, **D**, Representative flow cytometry cell cycle data in DAOY cells in response to 100 nM CPT (**C**) and 6.2 µM olaparib (**D**) at 0, 6, 12, or 24 h. The percentage of highly replicating cells is annotated. **E**, **F**, Representative immunoblots of whole cell lysates from the indicated DAOY cells treated with 100 nM CPT (**E**) and 6.2 µM olaparib (**F**). Blots were probed with the indicated antibodies.

Figure S5

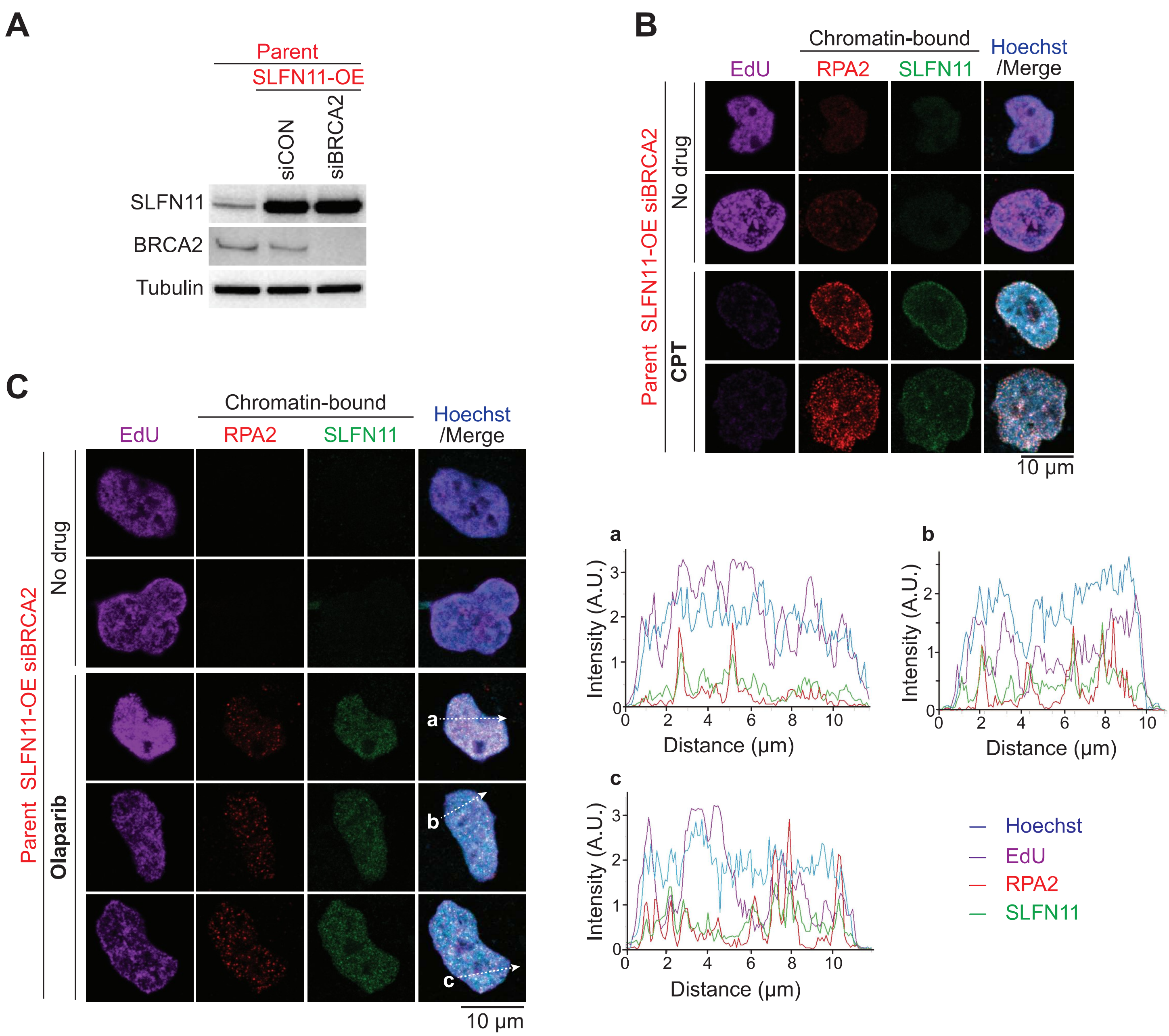

Figure S5. SLFN11 recruited behind a fork does not block replication. **A**, Representative immunoblots of whole cell lysates prepared from the indicated DAOY cells. Blots were probed with the indicated antibodies. OE: overexpression. **B**, **C**, Representative confocal microscopy images; EdU (purple), Hoechst (blue), chromatin-bound RPA2 (red), and SLFN11 (green) in the indicated DAOY cells. Cells were treated with or without 100 nM CPT for 6 h (**B**) and 10 μM olaparib for 12 h (**C**). Representative tracings of the distribution of signals along the white dashed arrow (**a**, **b**, and **c**) are shown in the merged panel.
